# Draft Genome Sequences of Two Escherichia albertii Isolates Collected from Healthy Pets in Switzerland

**DOI:** 10.1128/mra.01356-22

**Published:** 2023-02-23

**Authors:** Michael Biggel, Karen Barmettler, Andrea Treier, Francis Muchaamba, Roger Stephan

**Affiliations:** a Institute for Food Safety and Hygiene, Vetsuisse Faculty, University of Zürich, Zürich, Switzerland; University of Southern California

## Abstract

Here, we report the genome sequences of two Escherichia albertii isolates recovered from a healthy dog (KBD171i) and cat (KBK128i) in Switzerland in 2022. The genome sizes of KBK128i and KBD171i were 4.7 Mbp and 4.9 Mbp, respectively.

## ANNOUNCEMENT

Escherichia albertii is a zoonotic pathogen of the *Enterobacteriaceae* family and has sporadically been associated with human gastroenteritis (1–4). Its prevalence, distribution, and reservoirs are not yet clearly defined (5). In this study, we screened 100 healthy pets in Switzerland for potential carriage of *E. albertii*. Fecal samples from dogs (*n* = 51) and cats (*n* = 49) were taken by the pet owners between October 2022 and November 2022. After being shipped to the laboratory, samples were incubated overnight at 42°C in *Enterobacteriaceae* enrichment (EE) broth (Becton Dickinson). Cultures were then streaked onto sheep blood agar (Difco Columbia blood agar base EH; Becton Dickinson) and incubated again overnight at 42°C. Colonies were washed off with 2 mL NaCl (0.85%) and 100 μL thereof was combined with 200 μL Gram-negative lysis buffer. Samples were heated for 50 min at 60°C, then incubated for 10 min at 99°C, and centrifuged (11,000 rpm for 2 min). Supernatants were tested for the presence of the *E. albertii*-specific cytolethal distending toxin (*Eacdt*) gene by PCR (6). For *Eacdt*-PCR positive samples, a loopful of the washed-off bacterial suspension was streaked onto xylose MacConkey agar plates (7) and incubated overnight at 42°C. One cat sample (2%) and three dog samples (6%) were PCR positive for *Eacdt*. For two of those samples (one cat and one dog sample), *E. albertii* isolates could be recovered. Matrix-assisted laser desorption ionization–time of flight mass spectrometry (MALDI-TOF MS; Bruker Daltronics) was used for preliminary genus identification. DNA was isolated from subcultures obtained from single colonies grown for 24 h at 37°C on sheep blood agar. Genomic DNA was extracted using the DNeasy blood and tissue kit (Qiagen). Libraries were prepared using the Nextera DNA flex library preparation kit (Illumina) and sequenced on the Illumina MiniSeq platform (2 × 150 bp). Illumina reads were trimmed with fastp v0.20.1 (8) and quality assessed using FastQC v0.11.9 (https://www.bioinformatics.babraham.ac.uk/projects/fastqc/). Draft assemblies were generated using SPAdes v3.14.1 (9) implemented in shovill v1.1.0 (https://github.com/tseemann/shovill). The genomes were annotated using the NCBI Prokaryotic Genome Annotation Pipeline (PGAP; v6.4) (10). Species and multilocus sequence typing were performed using rMLST (11) and mlst 2.22.0 (https://github.com/tseemann/mlst; Escherichia coli Achtman scheme). AMRfinder 3.10.24 was used for the detection of resistance genes (12), and ABRicate 1.0.1 (https://github.com/tseemann/abricate) (minimum coverage, 50%; identity, 70%) in combination with VFDB set B (13) was used for the detection of virulence genes. Default parameters were used for all software unless otherwise stated. This study was performed in accordance with the Swiss Animal Welfare Act (SR 455).

The draft assembly sizes of KBK128i (cat isolate) and KBD171i (dog isolate) were 4.7 Mbp and 4.9 Mbp, respectively. Further sequencing and assembly statistics are given in Table 1. The two isolates were typed as *E. albertii* sequence type 14139 (ST14139) and ST2700, respectively. Notably, out of 10 Escherichia spp. ST2700 genomes available on Enterobase (14) (accessed 23 December 2022), four represent dog isolates (obtained in Australia, Finland, or United States), suggesting a dog-associated lineage. KBK128i and KBD171i did not harbor acquired antimicrobial resistance genes or Shiga toxin-encoding genes, which have been described for some *E. albertii* strains.

**TABLE 1:** Sequencing data and assembly metrics

Isolate	Total no. of reads	Total read length (bp)	Mean coverage (×)	Genome size (bp)	No. of contigs	*N*_50_ value (bp)	GC content (%)	Genome accession no.	SRA accession no.
KBK128i	1,659,327	247,494,708	52	4,722,156	63	391,697	49.65	JAPYXT000000000	SRR22864269
KBD171i	2,208,017	329,691,960	67	4,896,591	83	558,702	49.47	JAPYXU000000000	SRR22864268

### Data availability.

Sequencing data were deposited in GenBank and the NCBI Sequence Read Archive (SRA) under BioProject accession number PRJNA879956. Individual accession numbers are listed in Table 1.
